# Relationship between Viscosity, Microstructure and Electrical Conductivity in Copolyamide Hot Melt Adhesives Containing Carbon Nanotubes

**DOI:** 10.3390/ma13204469

**Published:** 2020-10-09

**Authors:** Paulina Latko-Durałek, Rafał Kozera, Jan Macutkevič, Kamil Dydek, Anna Boczkowska

**Affiliations:** 1 Faculty of Materials Science and Engineering, Warsaw University of Technology, 02-507 Warsaw, Poland; rafal.kozera@pw.edu.pl (R.K.); kamil.dydek@pw.edu.pl (K.D.); anna.boczkowska@pw.edu.pl (A.B.); 2 Technology Partners Foundation, 02-106 Warsaw, Poland; 3 Faculty of Physics, Vilnius University, 10222 Vilnius, Lithuania; jan.macutkevic@gmail.com

**Keywords:** carbon nanotubes, hot melt adhesives, electrical conductivity, viscosity, microstructure

## Abstract

The polymeric adhesive used for the bonding of thermoplastic and thermoset composites forms an insulating layer which causes a real problem for lightning strike protection. In order to make that interlayer electrically conductive, we studied a new group of electrically conductive adhesives based on hot melt copolyamides and multi-walled carbon nanotubes fabricated by the extrusion method. The purpose of this work was to test four types of hot melts to determine the effect of their viscosity on the dispersion of 7 wt % multi-walled carbon nanotubes and electrical conductivity. It was found that the dispersion of multi-walled carbon nanotubes, understood as the amount of the agglomerates in the copolyamide matrix, is not dependent on the level of the viscosity of the polymer. However, the electrical conductivity, analyzed by four-probe method and dielectric spectroscopy, increases when the number of carbon nanotube agglomerates decreases, with the highest value achieved being 0.67 S/m. The inclusion of 7 wt % multi-walled carbon nanotubes into each copolyamide improved their thermal stability and changed their melting points by only a few degrees. The addition of carbon nanotubes makes the adhesive’s surface more hydrophilic or hydrophobic depending on the type of copolyamide used.

## 1. Introduction

Adhesive bonding is frequently used to join and repair lightweight thermoplastic or thermosetting matrix composite parts in the automotive and aircraft industry since it eliminates rivets, thus lowering the stress concentration and total weight of the final parts [1,2,3]. Nevertheless, the separation of two bonded composites by an electrically insulating layer formed by the polymeric adhesive is a real problem in terms of the lightning strike protection of the aircraft or automotive composite structures. Therefore, the idea is to use electrically conductive adhesives (ECAs) which strongly bond composites together while at the same time providing an electrical interconnection between them [4]. These double functions of ECAs are also desired in the electronics industry (interconnection of chips on printed circuit boards) and in the photovoltaic industry (assembly of the aluminum back surface field or shingled solar cells) and make them a promising solution for the replacement the traditional Pb–Sn solder alloys, much heavier than ECAs [5,6].

ECAs consist of a polymer matrix and electrically conductive filler or nanofiller. Depending on the type of filler and its concentration, ECAs can be divided into isotropic, anisotropic and non-conductive according to the percolation theory. Isotropic ECAs have a high content of conductive filler that exceeds the percolation threshold and they are able to conduct current in all directions (x, y, z), unlike anisotropic and non-conductive ECAs which are conductive in only one direction [7]. Silver in the form of flakes [8], powder [9], nanowires [10] or dendrites [11] is the main type of electrically conductive filler which for a long time has been used in ECAs due to its excellent electrical conductivity and thermal stability. However, the minimum content of silver necessary to achieve a sufficient level of electrical conductivity at which a decrease in polymer resistivity is observable, varies from 25 wt % up to even 80 wt % [12]. A higher amount of filler leads to a significant weight increase, increases the price of the adhesive and lowers the mechanical properties [13]. Hence, the research focused on decreasing the silver percentage by partially replacing it with alternative electrically conductive nanofillers or using these nanofillers alone. Studies frequently focus on such promising materials as carbon-based nanofillers, e.g., graphene, single-walled carbon nanotubes (SWCNTs), multi-walled carbon nanotubes (MWCNTs), reduced graphene oxide or carbon nanofibers, known to be highly conductive and lightweight. The amount of these nanofillers required for electrically conductive network formation, identified by percolation threshold, is much lower in comparison to the metallic particles, usually between 1 and 3 wt % [14]. Compared to other types of carbon nanofillers, MWCNTs are characterized by excellent electrical properties (8 × 10^−6^ ÷ 20 × 10^−6^ Ωm), thermal conductivity (λ = 6600 W/mK at 100 K), a high Young’s modulus (1.7–2.4 TPa) and tensile strength (100 GPa) as well as low cost (100 EUR/1 kg) [15,16]. 

The polymers commonly used as the matrix for ECAs are those based on thermosets such as acrylic, epoxy, urethane, cyanoacrylate or silicone available as one- or two-component liquid systems. Epoxy resin, the most popular matrix for the adhesive, has been doped with various types of carbon nanofillers using the three-roll mill technique by Lopes et al. [6]. The greatest decrease in epoxy resistivity—achieved for SWCNTs, MWCNTs and exfoliated graphite—was higher than that of graphite, carbon fiber and nanofibers. In other work, epoxy resin was mixed with MWCNTs and the highest electrical conductivity was 10^0^ S/m, much lower than that obtained at 80 wt % silver [17]. Enhancing the electrical conductivity of the ECAs containing carbon nanofillers is realized by increasing their content up to even 50 wt % reported for reduced graphene oxide (r-GO) in epoxy resin applying ultrasonic technique [18]. The highest achieved volume electrical conductivity was 3.4 × 10^−8^ S/m, or two orders of magnitude lower than that determined for the composites containing only 2 wt % of graphene [19]. It is associated with the strong effect of the type of carbon nanofiller on the electrical conductivity, mainly its aspect ratio, purity, surface area and the presence of functional groups described previously for many thermosets and thermoplastic polymers [17,20]. Furthermore, it was shown that the viscosity, crystallinity content, polarity of the polymer as well as the mixing technique and applied conditions affect the dispersion and distribution of the nanofillers in the polymer matrix. Carbon nanofillers are synthesized in the form of strongly connected agglomerates which must be destroyed during processing to a homogenous dispersed state. The remaining agglomerates, and the orientation and alignment of the dispersed nanofiller are responsible for the level of electrical conductivity achieved in the polymer composites [21,22].

In order to increase the electrical conductivity, carbon nanofillers can be modified by metallization with silver, copper, or nickel particles by chemical reactions. Acrylic-based adhesives containing 2 wt % MWCNT metallized with silver resulted in a volume electrical conductivity of about 2.8 × 10^−6^ S/m [23], an insufficient value for application as ECAs. Therefore, considerable effort is being made put to form hybrid ECAs by mixing micro and nanofillers together due to their confirmed synergic effect caused by changing the contact resistance inside the electrical network [24]. Marcq and co-workers [25] found that epoxy adhesives doped with silver and SWCNTs, DWCNTs (double-walled carbon nanotubes) and MWCNTs do indeed possess higher electrical conductivity than adhesives containing only 25 wt % silver flakes. Similar improvement of the electrical conductivity was described for epoxy adhesives mixed with micron silver flakes, nano silver spheres and treated CNTs in comparison to epoxy resin containing only silver flakes [26]. A synergic effect occurring between fillers with different morphologies was also described for acrylate resin mixed with silver-plated graphene, leading to the greatest electrical conductivity of 4.8 × 10^2^ S/m at 40 wt % content of the hybrid filler [27].

The fabrication of ECAs from thermosets, especially with the hybrid nanofillers, involves many steps, chemical reactions, hazardous solvents and their curing process requiring the addition of catalysts or initiators, UV light or heat which make the process complicated and lengthy [28]. Besides, the incorporation of carbon nanofillers is limited to 3 wt % by an extremely high increase in the viscosity which makes the manufacturing process difficult and affects the quality of the composites [29]. Therefore, it seems much easier to use thermoplastic non-reactive hot-melt adhesives (HMAs), solvent-free and environmentally friendly polymeric materials available in a solid state in the form of pellets, ropes, sticks or blocks. They can be applied to the surface by using a hot gun or in the form of thin films or fabrics. When HMAs are melted at a temperature above their melting point (usually in the range of 150–200 °C) they become a molten liquid which solidifies at room temperature. Because HMAs thicken rapidly, they are frequently used in those processes where manufacturing time is important and solvents are not desired. Moreover, they offer a high mechanical strength to the substrate without the need for special surface preparation. In addition, commercial HMAs offer a high range of properties together with fairly low prices, safe storage and operation. In comparison to the liquid adhesives like epoxy or urethanes which become solidified via curing or crosslinking reaction, HMAs do not form three-dimensional structures when they solidify and can thus be termed as non-structural adhesives. Nowadays, they are used in the packaging industry (bottle labeling, tapes, carton sealing), the automotive industry to bond plastic parts (rear tail gate, bumper, aesthetic skin), wood-based materials (parquet floor), book binding, temporary attachment (coupons, instructions), shoe manufacturing (insole bonding, tongue fixing), textile bonding (diapers, napkins) or as wearable electronic devices and lightweight constructions [2,30,31]. 

HMAs are complex in composition, therefore they cannot be classified as typical thermoplastic polymer but rather as blends. In our previous work, we attempted to identify all the components of the copolyamide-based HMAs using a nuclear magnetic resonance but we could ascertain their structure only partially [32]. The main component in HMAs is the thermoplastic polymer which solidifies upon cooling and provides the mechanical strength of the bond. Homopolymers or copolymers such as polyolefins, ethylene-vinyl acetate copolymers (EVA), polyurethanes, polyamides, copolyamides, styrene block copolymers (SIS, SEBS) or polyesters are mainly used in HMAs. Low molecular weight resin or tackifiers control the viscosity of the system, adjust its glass transition temperature and provide the adhesive properties. Waxes can decrease the viscosity and enhance the crystallization rate resulting in higher setting speed. Finally, there are also several other specific fillers and modifiers that can be used for the improvement of thermal stability and shelf life [31,33,34]. The doping of thermoplastic HMAs with electrically conductive fillers or nanofillers for their application as ECAs is less popular than for thermosets. Polyurethane-based HMAs containing MWCNTs or graphene were extensively studied by Santamaria and co-workers [35,36,37,38,39]. Composites were prepared by mixing HMA powder with up to 6 wt % of MWCNTs or graphene using a small scale twin screw extruder. They reported that the addition of MWCNTs or graphene did not significantly change properties such as the melting point, viscosity, crystallization or tackiness which could limit their use as ECAs. The analyzed electrical conductivity of the adhesives after cooling reached the value of 6 × 10^−2^ S/m which is higher than that described in the literature for ECAs based on thermosets. Similar electrical properties (10^−2^ S/m) were found for polyolefin HMAs mixed with 5 wt % MWCNT using the same direct melt–mixing approach [40]. Authors found that the integration of MWCNTs in polyolefin HMAs resulted in the improved adhesion of the bonded joints, however, the significant increase in melt viscosity made it impossible to apply adhesives containing more than 3 wt % MWCNT. Cecen et al. examined silver-coated wollastonite fibers as a conductive filler for the EVA copolymer [41]. The percolation threshold was found at 8 vol% of the filler, much higher than for the carbon nanofiller, and at 29 vol% the electrical conductivity reached a value of 1.8 × 10^5^ S/m. Unfortunately, such high content of the conductive filler significantly decreased the adhesive properties in comparison to pure EVA. In the other works, HMA based on EVA was mixed with polypyrrole as a conductive filler which at 30 vol% resulted in the electrical conductivity of app. 1 × 10^2^ S/m and a 15–20% of improvement of the adhesive properties [42]. 

The aim of this study was to characterize the new type of the ECAs based on copolyamide HMAs and MWCNTs. Since HMAs are complex in their structure, the idea was to test four types of these copolyamides to analyze the effect of their melt viscosity on the dispersion and distribution of MWCNTs. Previously, we analyzed the percolation threshold in two types of copolyamide HMAs and found it to be below 3 wt % [32]. Therefore, in that work, copolyamides were doped with 7 wt % of MWCNTs using a half-industrial extruder machine allowing to obtain the percolated network. The examination of their electrical, thermal and adhesive properties allowed for finding the relationship between the viscosity of pure copolyamides, the state of MWCNT dispersion obtained and the properties of the final HMAs containing electrically conductive nanofiller. 

## 2. Materials and Methods 

For this study, 4 types of thermoplastic copolyamides (coPAs) belonging to the group of HMAs were provided by EMS Griltech from Switzerland. According to the producer, they consisted of randomly arranged segments of PA6 and PA66 and differed in their properties as shown in Table 1. The conductive nanofiller used was MWCNTs with the trade name NC7000 from Nanocyl, Sambreville, Belgium) synthesized by catalytic carbon vapor deposition process. The average diameter of a MWCNT is 9.5 nm, length 1.5 µm and with purity >95%. All coPAs were mixed with 7 wt % of MWCNT using a half industrial line using a twin co-rotating screw industrial extruder by Nanocyl under the same processing conditions: an extrusion temperature of 200 °C and a rotational speed of 200 rpm. The neat coPAs and their masterbatches were dried in a vacuum oven at 80 °C for 12 h before further processing.

Rheology measurement was performed on an ARES rheometer (Rheometric Scientific Inc., TA Instruments, New Castle, DE, USA) using a parallel plate geometry. The samples with a diameter of 1.5 mm and a thickness of 2 mm were prepared using a HAAKE^TM^ Mini Jet Pro Piston Injection Molding System (ThermoScientific, Karlsruhe, Germany). Firstly, the linear elastic range was determined by conducting the amplitude sweep test of the materials. From the obtained graph, the amplitude strain was chosen as the highest value just before the moduli decreasing. Afterwards, the stress-controlled dynamic oscillatory test of neat coPAs and their masterbatches with MWCNTs was performed at 180 °C with a frequency sweep in the range of 0.1–100 Hz. 

The macrodispersion of MWCNTs in the masterbatches was analyzed using a light transition microscope (Biolar-PL, Polskie Zakłady Optyczne, Warsaw, Poland). Samples for the test in the form of slides with a thickness of 2–3 μm were cut directly from the masterbatch pellets using an ultramicrotome (EM UC6, Leica, Vienna, Austria). The macrodispersion of MWCNTs was analyzed from several micrographs with image software (ImageJ version 1.52a) by the exclusion of those agglomerates with a diameter lower than 1 µm. Area ratio (A_A_) understood as the percentage of MWCNT agglomerates was calculated by dividing the area of all agglomerates by the total area. 

Characterization of MWCNT dispersion and arrangement in nanometer scale was analyzed using a transmission scanning electron microscope (HR STEM S5500, Hitachi, Krefeld, Germany) at the voltage of 30 kV. For this, thin slides of 80–90 nm were cut directly from the masterbatch pellets using an ultramicrotome (EM UC6, Leica, Vienna, Austria). The cutting process was carried out with diamond knives that are suitable for trimming and sectioning, at a temperature of 100 °C and with a cutting speed of 1 mm/s. 

Broadband dielectric spectroscopy of the studied materials was performed at room temperature using a LCR HP4284A meter (Keysight Technologies, Santa Rosa, CA, USA). The equivalent electrical circuit was selected as the capacitance and the tangent of losses connected in parallel. From these quantities, according to the planar capacitor formula, the complex dielectric permittivity Ɛ* of the studied materials was calculated. The relation σ = 2πνε_0_ε^*^ was used for the determination the electrical conductivity σ, where ω = 2πν and ν is the frequency of electromagnetic waves. To minimize the contact resistance silver paste was applied and the amplitude of the electric field was chosen as 1 V. Measurements under such conditions make it possible to reach the best signal to noise ratio in comparison with measurements at lower voltages and to avoid the nonlinear effects observed at higher voltages.

The volume DC electrical conductivity of the masterbatches containing MWCNTs was carried using the Keithley 6221/2182A measuring set (Cleveland, OH, USA), equipped with copper electrodes. Samples for the test were produced by thermo-pressing the dried pellets into rectangular shapes with the dimensions of 70 mm × 10 mm and thickness of around 1.5 mm. For each masterbatch, 5 samples were tested within the current range from 1 to 200 nA. To compensate for thermoelectric effects, measurements were made in the delta mode, using the four-point method.

Thermal stability of the materials expressed by the degradation temperature occurs at 2% (T_2%_) and 5% (T_5%_) weight loss, and by maximum peak (T_d_) was designated by thermogravimetric analysis (TGA) using a TGA Q500 (TA Instruments, New Castle, DE, USA). For that 10 ± 0.2 mg samples were placed in an aluminum crucible and heated from 0 °C to 1000 °C with a heating rate of 10 °C/min. The atmosphere was nitrogen with a flow rate from 10 to 90 mL/min. 

Thermal properties of all materials were examined using a Q1000 Differential Scanning Calorimeter (TA Instruments, New Castle, DE, USA) by placing the samples with a weight of 8.5 ± 0.2 mg in an aluminum hermetic pan under a nitrogen atmosphere. The applied program was heat–cool–heat from −60 °C to 250 °C with a scan rate 10 °C/min and the obtained curves were analyzed using TA Universal Analysis 2000 software version 4.5A. The glass transition temperature (T_g_), melting point (T_m_) and enthalpy of melting (ΔH_m_) were determined from the heating curves, while the crystallization temperature (T_c_) and the enthalpy of crystallization (ΔH_c_) were found in the cooling curve. Due to the lack of data on the enthalpy of melting of 100% crystalline coPAs, the crystallinity content was not calculated.

Wettability properties (static contact angle (SCA), surface energy (SE)) were measured by a contact angle measurement system (Data Physics GmbH OCA 15, Filderstadt, Germany). All angles of each sample were measured at least three times across the sample surface using the sessile drop method, by dispensing 3 μL (SCA), of deionized water on the sample’s surface. The round samples for the wettability test were made by a HAAKE^TM^ Mini Jet Pro Piston Injection Molding System (ThermoScientific, Karlsruhe, Germany) with a diameter of 1.5 mm and thickness of 2 mm, the same as for the rheological test. For both pure coPAs and their masterbatches, the injection parameters were as follows: barrel temperature—220 °C, mold temperature—40 °C, injection pressure—600 bar, injection time—8 s, post pressure—500 bar and post-pressure time—8 s. 

## 3. Results and Discussion

**Figure 1:**
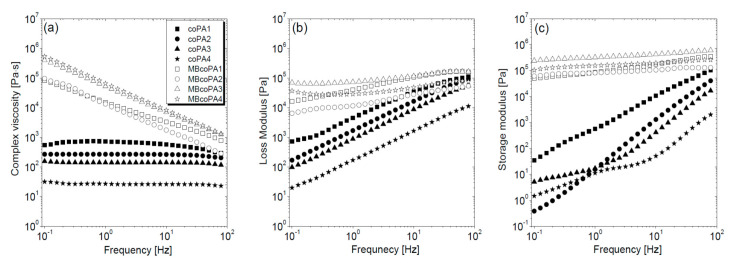
(**a**) Complex viscosity; (**b**) loss modulus G’’ and (**c**) storage modulus G’ in the frequency dependence for the neat coPAs and their masterbatches containing 7 wt % multi-walled carbon nanotube (MWCNT).

HMAs are characterized by their application temperature which for the selected coPAs is between 160 °C and 220 °C, therefore the rheological test was performed at 180 °C. Unfilled coPAs differ in viscosity which is the lowest for coPA4, followed by coPA3, coPA2 and the highest for coPA1, as presented in Figure 1a. All coPAs behave as Newtonian liquids since the frequency applied has no effect on the complex viscosity [43]. Unlike coPAs, masterbatches which exhibit a strong shear thinning behavior causing a decrease in their viscosity have been described for many thermoplastic polymers filled with CNTs [29]. In comparison to neat coPAs, the viscosity increases by about 4–5 orders of magnitude for the low viscosity coPA3 and coPA4 and about three orders of magnitude for the more viscous coPA1 and coPA2. The analysis of the storage (G’) and loss (G’’) modulus provides information about the elastic and viscous properties of polymers, respectively [44]. Figure 1b,c show the sharp growth of G’ and G’’ as the frequency increases. Because G’’ is higher than G’, coPAs behave more like viscous liquid than elastic. The character of both modulus curves changes in the presence of 7 wt % MWCNT in an almost linear manner across the whole frequency range. For the studied coPA masterbatches the effect of MWCNT addition is visible through an increase of about 5–6 and 3–4 orders of magnitude for G’ and G’’, respectively. The jump in G’ and G’’ is caused by the interaction between the polymer macromolecules and CNTs and disturbing the macromolecule chains’ movement. Since the MWCNTs used do not contain any functional groups which promote the formation of covalent bonds with the polymer chains, MWCNTs are connected with coPA chains by van der Waals forces [45]. However, this scenario is favored in low viscosity polymers because MWCNTs can enter easily between the macromolecule chains. The opposite is true in more viscous coPAs, where MWCNT dispersal is hampered due to the high degree of chain entanglement. Therefore, more hydrogen bonds are formed between the polymer chains themselves than between the polymer and MWCNTs [46]. Hence, the differences in the MWCNT dispersion should be expected. Moreover, it seems that the highest compatibility expressed by the value of G’ and G’’ occurs for the coPA3 matrix. Rheological analysis confirms that these types of HMAs are a good polymer matrix for MWCNTs because there is a clear change in the viscoelastic properties of coPAs, and at high concentration such as 7 wt %, the nanofiller forms a percolated structure. The obtained values of both moduli (~10^5^ Pa) are the same as those determined for HMAs based on polyurethane [39].

**Figure 2:**
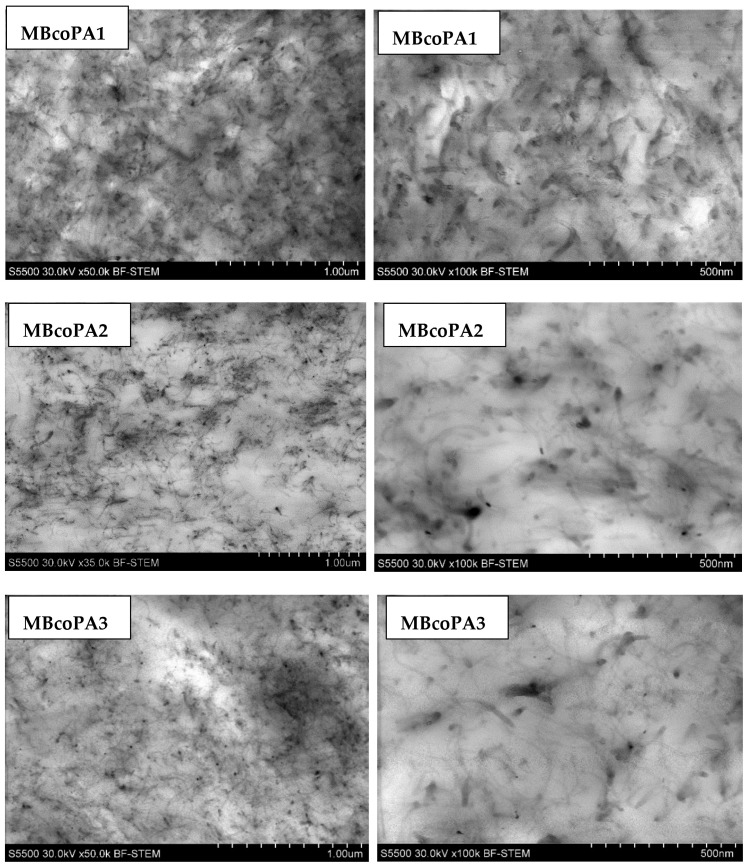

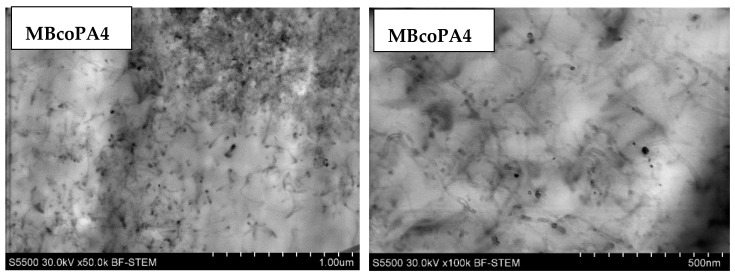
HR-STEM images of the microstructure of the copolyamide masterbatches containing 7 wt % MWCNT.

In order to fully realize the potential of CNTs, the key step is to disperse the nanofiller uniformly in the polymer matrix. One of the methods of achieving this is twin screw extrusion where due to high shear force the pristine MWCNT agglomerates are effectively broken [47]. On the one hand, the less viscous polymer allows for a better CNT dispersion due to the easy infiltration of the nanofillers, whereas when the polymer is more viscous, a higher shear force occurs during extrusion, leading to better CNT dispersion [48]. To analyze the effect of the coPA melt viscosity on the MWCNT dispersion, four types of coPAs were mixed with 7 wt % MWCNT under the same extrusion conditions. Table 2 contains the micrographs of the masterbatches where black dots signify MWCNT agglomerates quantitatively expressed by their area ratio A_A_ (last column). It is seen that there is no linear dependence between the viscosity of coPAs and the number of MWCNT agglomerates. This is because the fewest agglomerates were found in coPA3, which has a medium viscosity. Indeed, for coPA1, which is the most viscous, the percentage ratio of agglomerates is lower than for coPA2 and coPA3, characterized by lower viscosity. This is consistent with the theory about the positive effect of the high shear force on the CNT agglomerate breakage, also confirmed by the lowest diameters (<40 µm) of MWCNT agglomerates in coPA1 (see column 3, Table 2). Conversely, in coPA3, where A_A_ is the lowest, agglomerates have higher diameters because the shear force during extrusion is lower than in the case of coPA1. Despite the significant difference in the melt viscosity, masterbatches of coPA2 and coPA4 have similar A_A_ and agglomerate diameters. The analysis of the nanofiller macrodispersion for coPA masterbatches showed that they are uniformly distributed in the polymer matrix. The homogenous distribution of MWCNTs in each coPA was also confirmed by the images given by a high-resolution microscope (Figure 2). From the presented micrographs it is shown that MWCNTs were not arranged or oriented in any specific direction.

**Figure 3:**
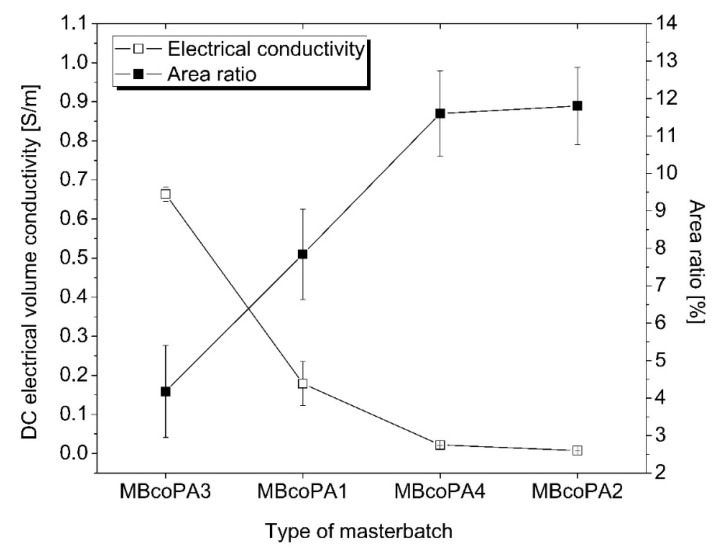
Dependence between the volume electrical conductivity and A_A_ for the studied masterbatches.

The electrical conductivity of the coPA HMAs is the main property for their final application as ECAs. It is known that only a uniform dispersion and distribution of nanofillers such as MWCNTs results in high electrical properties. Figure 3 presents the average values of the volume electrical conductivity for four types of masterbatches, with the highest recorded for MBcoPA4, followed by MBcoPa1, MBcoPA4 and the lowest for MBcoPA2. These results were correlated with the calculated A_A_ and it is clearly shown that electrical conductivity increases when A_A_ decreases. This means that MBcoPA3 possessed the highest electrical conductivity equal to 0.67 S/m because the percentage ratio of MWCNT agglomerates was the smallest (4.18%). The values of electrical volume conductivity achieved for MBcoPA3 are higher than those reported for the other types of ECAs containing carbon nanofillers but lower than those for ECAs with silver flakes, as listed in Table 3. Despite this, the weight of the carbon nanofillers used was less than that of the metal fillers, which is highly desired in ECAs. Assuming the price of coPA to be 10 EUR/1 kg, the price of MWCNTs to be 100 EUR/1 kg and the price of the silver flakes, 1000 EUR/1 kg, the cost of the filler in coPA adhesive will be 43% for MWCNTs and almost 99% for silver. 

MBcoPA1 and MBcoPA3 were about one order of magnitude higher than for polyolefin HMAs containing 5 wt % of MWCNTs [49] and polyurethane ECAs mixed with 6 wt % of graphene [39]. In Table 3, there is a comparison of the electrical conductivity of the different ECAs. 

**Figure 4:**
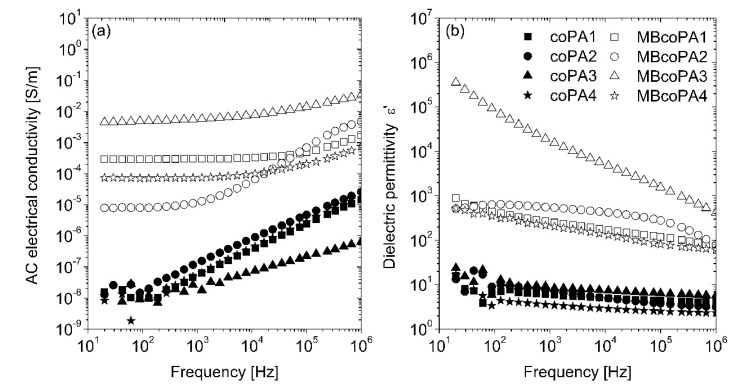
Comparison of: (**a**) the real part of the electrical conductivity; and (**b**) the real part of the dielectric permittivity for the neat coPAs and their masterbatches containing 7 wt % MWCNT.

The frequency dependence of AC electrical conductivity and dielectric permittivity for pure coPAs and their masterbatches containing 7 wt % MWCNT is shown in Figure 4a,b. As expected, pure coPAs have rather low (around 10^−8^ S/m) electrical conductivity at low frequency and even 10^−5^ S/m at high frequency. Moreover, no frequency independent (DC) conductivity is observed in the conductivity spectra of pure coPAs, therefore the AC electrical conductivity is related to some dielectric relaxation rather than the electrical transport [50]. The dielectric permittivity (ɛ’) is around 10 at the whole frequency range and it is much higher than that reported for the typical homopolymers like PA6 and PA66 [51]. Such discrepancies as well as the slight differences in the dielectric properties presented for unfilled coPAs may be associated with the HMA complex formulation (resin, tackifier, wax, etc.) affecting their polarity. The incorporation of 7 wt % MWCNT into coPAs resulted in a significant increase in the electrical properties and dielectric permittivity observed for each masterbatch. The electrical conductivity rose by 3 to 6 orders of magnitude depending on the type of coPA, up to 10^−2^ S/m. At low frequencies, conductivity curves have a plateau that corresponds to the DC electrical conductivity of the electrically percolated network [14]. Determined at 129 Hz, electrical conductivity was the lowest for MBcoPA2 (8.02 × 10^−6^ S/m), followed by MBcoPA4 (7.07 × 10^−5^ S/m), MBcoPA1 (2.95 × 10^−4^ S/m) and with the highest for MBcoPA3 (4.87 × 10^−3^ S/m). These results are consistent with the macrodispersion of MWCNTs presented in Table 2 and DC electrical conductivity results shown in Figure 3. Conductivity spectra obey the Almond–West power law:

(1) $$\sigma \left(\omega \right)=\sigma_{DC}+{\left(\frac{\omega }{\omega_{cr}}\right)}^{s}$$

where *σ_DC_* is the *DC* conductivity, *ω_cr_* is the critical frequency at which the conductivity *σ*(*ω*) from the *DC* plateau, and *s* is the parameter. From Figure 4a, it is possible to conclude that the behavior of critical frequency is correlated with the behavior of the *DC* conductivity, with no correlation with behavior of parameter *s.* A factor which is important for the frequency-dependent conductivity *σ*(*ω*) is the electron transport not only across the whole sample but also inside some conductive clusters, if the electron flight time τ inside the cluster is smaller than the reciprocal electromagnetic wave frequency 1/2π*ω*. Thus, bigger aggregates are related with smaller critical frequencies, while parameter *s* is related with the distribution of aggregates’ size [52] (Figure 4a and Table 2). 

The incorporation of MWCNTs led to an increase in the dielectric permittivity, by about two orders of magnitude for masterbatches based on coPA1, coPA2 and coPA3, and much more (10^6^) for coPA3. The behavior of dielectric permittivity at low frequencies (for example 129 Hz) is correlated with the *DC* conductivity behavior, except in sample MBcoPA2. The dielectric permittivity of this sample is higher than the dielectric permittivity of samples MBcoPA1 and MbcoPA4. Such mismatch in the behavior of dielectric permittivity and electrical conductivity was already explained by the difference in distributions of relaxation times and distributions of agglomerate size [52]. Indeed, the distribution of agglomerate size is much broader for sample MBcoPA2 than for samples MBcoPA1 and MBcoPA4 (see Table 2). Together with the frequency, the dielectric permittivity decreased as was reported for thermoplastics [53], elastomers [54] and thermoplastic elastomers [55] containing CNTs. 

**Figure 5:**
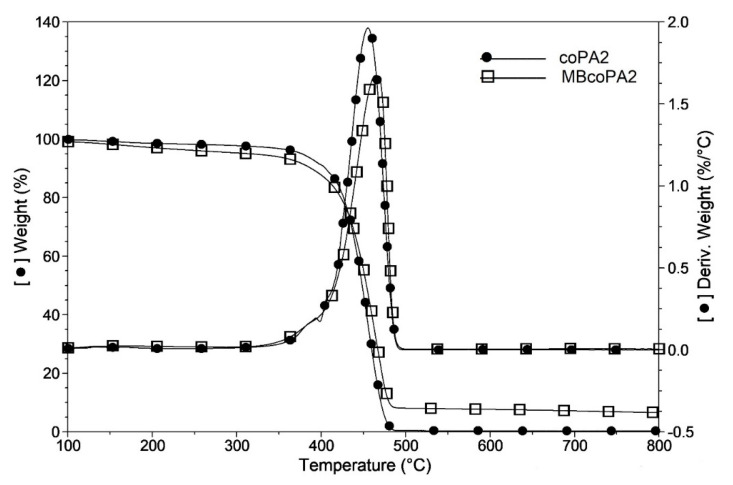
The example TGA curve for the neat coPA2 and its masterbatch containing 7 wt % MWCNT.

It has been observed previously using the TGA method, that in the presence of CNTs, the flux of degradation products is hindered and delays the start of the degradation process of the polymer [56]. The influence of the addition of MWCNTs on the coPAs’ thermal stability was examined by TGA and the results are presented in Table 3. The example TGA curves for coPA and the masterbatch are presented in Figure 5. It is seen that for all pure coPAs at 2% weight loss, the decomposition starts below 200 °C with the lowest temperature T_2%_ for coPA3. In the presence of 7 wt % MWCNT, the decomposition temperature (T_2%_) rises the most for MBcoPA1 and for MBcoPA2 by about 103 °C and 88 °C, respectively. Interestingly, for MBcoPA3 and MBcoPA4, the increase is only a few degrees. For them, higher increase in the decomposition temperatures was determined at 5 wt % weight loss, about 23 °C (MBcoPA3) and 15 °C (MBcoPA4). It is worth noting that the decomposition process in pure coPAs starts at a much lower temperature than that reported for the typical PA6 and PA66 homopolymers which decompose at 350 °C [57]. The maximum rates of weight loss for pure coPAs occurs at 450 °C and this temperature raises slightly (6–17 °C) in the presence of 7 wt % MWCNT. It seems that such a high amount of MWCNTs has a smaller effect on the thermal stability of the polymer than the lower concentration. The PA6 composites mixed with only 0.5 wt % MWCNT caused the shift of about 70 °C in the temperature decomposition [58].

**Figure 6:**
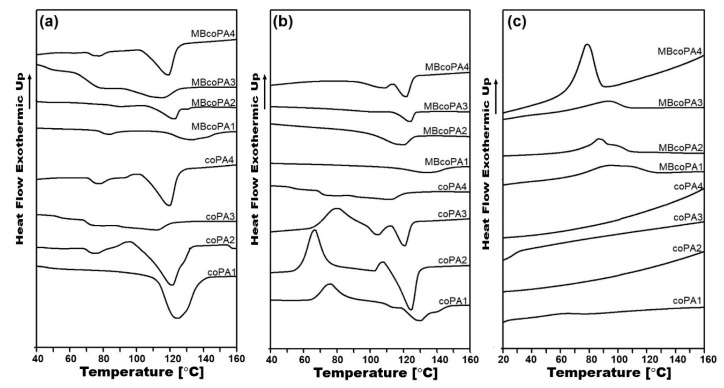
(**a**) First heating curves; (**b**) second heating curves; and (**c**) cooling curves for the unfilled coPAs and their masterbatches containing 7 wt % MWCNT.

The inclusion of CNTs in the polymer alters the nucleation process, resulting in formation of a crystalline phase which in turn can affect the electrical properties of the nanocomposites. The DSC method was used to determine the thermal properties of the materials listed in Table 4 and collected in Figure 6. On the post-processing, the first heating curves (Figure 6a), two peaks from the glass transition (T_g_) and the melting point (T_m_) were detected. For the unfilled coPA1 and coPA3, T_g_ occurs at a lower temperature of around 50 °C, while for coPA2 and coPA4, at a higher temperature of around 70 °C. Because selected coPAs consist of the PA6/PA66 copolyamide, the lower T_g_ is probably associated with PA6 segments; in turn, the higher T_g_ temperature comes from PA66 segments [59]. The addition of 7 wt % MWCNT shifts the T_g_ peak towards higher values since well dispersed MWCNTs hamper the mobility of the polymer chains [60]. Melting points for pure coPAs are much lower than for typical PA6 and PA66 homopolymers, between 110 and 130 °C, as visible during the first and second heating curves (Figure 6a,b). Due to the complex formulation of coPAs, the melting peak is broad, especially for coPA3, meaning that the crystal phases are not homogenous. Moreover, for all coPAs except coPA3, there is clear evidence for the cold crystallization visible as an exothermic peak and it can be associated with the too slow crystallization process. After the addition of 7 wt % MWCNT, the melting peak is shifted but only by a few degrees (2–6 °C) meaning that their application temperature (180 °C according to Materials Safety Data Sheet) will remain unchanged. The nucleating role of MWCNTs is also confirmed by the cooling curves presented in Figure 6c. Interestingly, pure coPAs do not possess any thermal processes. However, for 7 wt % MWCNT masterbatches, broad peaks appear at the maximum of around 85 °C for MBcoPA1, MBcoPA1 and MBcoPA3 and with a sharper peak for MBcoPA4 with the maximum at 92 °C. The sharper the peak, the more perfect crystals are formed which come from one type of polyamide segment [61]. In comparison to typical homopolymers in which the crystallization is more clear due to their homogenous structure, in HMAs, the crystallization process may be disturbed by the presence of various components [59,60,61]. The formation of the new crystal phase can be noticed by the changes in the enthalpy of melting, ΔH_m_. There is the same decreasing in the enthalpy of melting determined at the first and second heating after the incorporation of 7 wt % MWCNT. At the first heating, the most for the MBcoPA1 was about 31.8 J/g, then for coPA1 about 14.7 J/g and for coPA2 and coPA4, it was 10.4 J/g and 8 J/g, respectively. During the second heating, these differences were smaller, with only a few J/g except for MBcoPA2 where the ΔHm decreased by about 21.4 J/g. 

In order to see the differences in the adhesion properties of the pure coPAs and their masterbatches with 7 wt % MWCNT, the analysis of the contact angle and surface energy was performed. According to the results collected in Table 5, among all the studied coPAs, coPA3 had a significantly higher contact angle and lower surface energy than the other coPAs. The differences in the wettability of the selected coPAs were related to their specific compositions; especially the type and content of the tackifier [62]. Because CNTs change the surface properties of the polymers, the wettability of the nanocomposites will be not the same as of the pure coPAs. As shown in Table 4, the inclusion of 7 wt % MWCNT resulted in the greatest decrease in the contact angle for MBcoPA2 by about 31°, followed by a decrease of 19° for MBcoPA3 and the lowest for MBcoPA1 by about 7°. The calculated surface energy for these masterbatches increased in the same order as an effect of the modification of the surface by the addition of MWCNTs. Yang at al. also reported an improvement of the hydrophilicity of the gutta-percha nanocomposites at 2 wt % MWCNT content [63]. It should be noted that the values of the contact angle in coPA1 and coPA2 as well as their masterbatches indicate the hydrophilic character of their surfaces, resulting in good adhesion and wettability. For coPA3, the contact angle was 99°, which means that this coPA was more hydrophobic, but in the presence of MWCNTs, the surface became hydrophilic since the contact angle decreased to 80°. This was the opposite to coPA4, in which the unfilled polymer was more hydrophilic but the addition of MWCNTs promoted the hydrophobic surface properties (contact angle >90°) associated with the poor adhesion and wettability.

## 4. Conclusions

The present paper describes a new group of electrically conducive adhesives fabricated by the melt-blending of thermoplastic hot melt adhesives based on coPAs and 7 wt % MWCNT. The selected coPAs have low, medium and high viscosity which together with the loss and storage modulus was increased by about 3–6 orders of magnitude as an effect of the strong interactions occurring between coPA macromolecules and MWCNTs. It was confirmed that viscosity has no effect the on the dispersion and distribution of MWCNTs in the polymer matrix. From microscopic images, the area ratio of the agglomerates calculated by ImageJ was the lowest for coPA3 having MVR = 350 (A_A_ = 4.18%) and the highest for coPA2 (A_A_ = 11.8%) and coPA4 (A_A_ = 11.6%) having MVR = 600 and 150, respectively. The correlation of the determined number of agglomerates with the measured electrical conductivity of the coPAs + 7 wt % MWCNT clearly presented an increase in the electrical conductivity value when A_A_ decreased. Therefore, the highest DC electrical conductivity was achieved for coPA3 + 7 wt % MWCNT; σ = 0.67 S/m and the lowest for coPA2 + 7 wt % MWCNT σ = 0.0076 S/m. These results are consistent with the AC electrical conductivity analyzed by dielectric spectroscopy which also showed an increase in the dielectric permittivity in the presence of MWCNTs. The addition of 7 wt % MWCNT to coPAs shifted the decomposition temperature towards higher values, especially for coPA1 and coPA2, to about 103 °C and 88 °C, respectively. However, the thermal stability increased by only a few degrees since high MWCNT content is not as effective as a low concentration. Similarly, the melting points of coPAs + 7 wt % MWCNT increased by a few degrees (2–6 °C) which, together with the changes in the enthalpy of melting, indicates the nucleation effect of MWCNTs. The adhesion properties analyzed by the measurement of the contact angle and the surface energy indicated that depending on the type of coPA used and its composition, they are more hydrophobic or hydrophilic. The addition of MWCNTs modifies the surface of the nanocomposites visible by the enhancement of hydrophobicity or hydrophilicity of the coPAs. Thermoplastic hot melt copolyamides containing MWCNTs are the example of the ECAs which can be used to join the composite structures together to provide the conductive interlayer required for lightning strike protection. 

## Figures and Tables

**Table 1:** Properties of thermoplastic copolyamides (coPAs) used in the study.

Designation	Trade Name	Melt Viscosity 160 °C/2.16 kg (Pa·s)	Melt Volume Rate 160 °C/2.16 kg	Melting Point (°C)
coPA1	Griltex^®^ 1330	1200	9	125–135
coPA2	Griltex^®^ 2A	600	18	120–130
coPA3	Griltex^®^ 1858	350	30	110–120
coPA4	Griltex^®^ 1566	150	70	115–125

**Table 2:** The comparison of MWCNTs’ macrodispersion in the used coPAs expressed by agglomerates diameters and the area ratio. MV = melt viscosity in (Pa·s); s = standard deviation.

Masterbatch Type	Optical Image	Histogram	Area Ratio (%)
MBcoPA1 MV = 1200	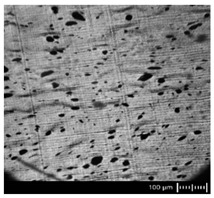	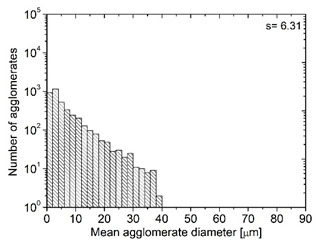	7.84 ± 1.21
MBcoPA2 MV = 600	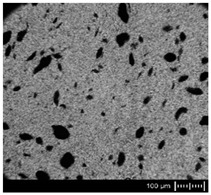	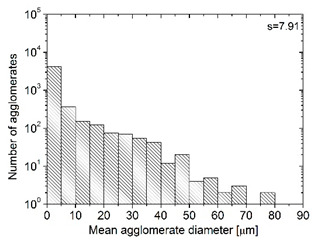	11.8 ± 1.03
MBcoPA3 MV = 350	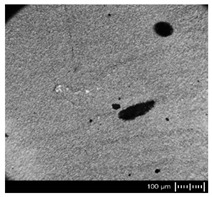	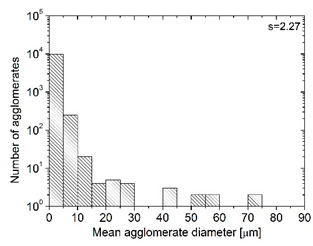	4.18 ± 1.23
MBcoPA4 MV = 150	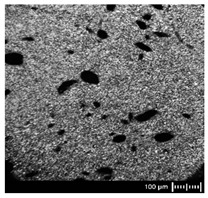	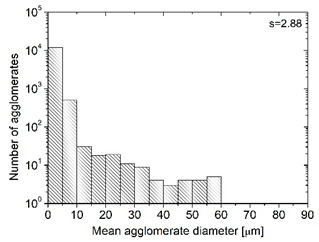	11.6 ± 1.14

**Table 3:** Electrical conductivity in different types of electrically conductive adhesives (ECAs).

Adhesive Matrix	Filler Type	Filler Content (wt %)	Electrical Conductivity (S/m)	Ref.
epoxy	silver flakes	70	10^2^	[11]
epoxy	reduced graphene oxide	50	10^−8^	[18]
epoxy	MWCNT	12	10^−1^	[17]
ethylene-vinyl acetate	graphite nanoplatelets	30	10^−5^	[50]
polyurethane HMA	graphene	6	10^−2^	[39]
polyolefin HMA	MWCNT	5	10^−2^	[40]
coPA3 HMA	MWCNT	7	0.67	this work

**Table 4:** Summary of the thermal analysis results. “---” means a lack of peak on the curve.

Material	TGA			DSC					
				First Heating			Second Heating		Cooling T_c_ (°C)
	T_2%_ (°C)	T_5%_ (°C)	T_d_ (°C)	T_g_ (°C)	T_m_ (°C)	ΔH_m_ (J/g)	T_m_ (°C)	ΔH_m_ (J/g)	
coPA1	188	339	455	46.1	130	64.1	128	31.8	---
MBcoPA1	291	379	461	50.1	133	32.3	133	29.0	92.7
coPA2	184	337	455	70.6	121	51.9	124	40.4	---
MBcoPA2	272	376	464	85.5	124	37.2	126	19.0	86.4
coPA3	171	271	443	52.8	111	25.1	110	16.8	---
MBcoPA3	178	294	457	70.5	116	14.7	116	15.3	84.8
coPA4	196	346	447	72.5	120	31.4	121	25.9	---
MBcoPA4	201	361	464	72.7	122	23.4	122	25.8	87.1

**Table 5:** Variation in the contact angle and surface energy for the pure coPAs and their masterbatches.

Material	Average Contact Angle (°)	Average Surface Energy (mN/m)
coPA1	85 ± 1.5	34.02 ± 0.005
MBcoPA1	78 ± 0.6	36.61 ± 0.004
coPA2	83 ± 5.0	33.44 ± 0.002
MBcoPA2	52 ± 3.0	52.81 ± 0.004
coPA3	99 ± 4.2	23.72 ± 0.005
MBcoPA3	80 ± 3.5	35.45 ± 0.006
coPA4	88 ± 3.1	30.43 ± 0.003
MBcoPA4	96 ± 0.6	25.92 ± 0.004
